# Transposon dynamics and the epigenetic switch hypothesis

**DOI:** 10.1007/s11017-021-09548-x

**Published:** 2021-12-17

**Authors:** Stefan Linquist, Brady Fullerton

**Affiliations:** Department of Philosophy, University of Guelph, Guelph, ON Canada

**Keywords:** Epigenetic inheritance, Transposable elements, Function concepts, Philosophy of genomics

## Abstract

The recent explosion of interest in epigenetics is often portrayed as the dawning of a scientific revolution that promises to transform biomedical science along with developmental and evolutionary biology. Much of this enthusiasm surrounds what we call the epigenetic switch hypothesis, which regards certain examples of epigenetic inheritance as an adaptive organismal response to environmental change. This interpretation overlooks an alternative explanation in terms of coevolutionary dynamics between parasitic transposons and the host genome. This raises a question about whether epigenetics researchers tend to overlook transposon dynamics more generally. To address this question, we surveyed a large sample of scientific publications on the topics of epigenetics and transposons over the past fifty years. We found that enthusiasm for epigenetics is often inversely related to interest in transposon dynamics across the four disciplines we examined. Most surprising was a declining interest in transposons within biomedical science and cellular and molecular biology over the past two decades. Also notable was a delayed and relatively muted enthusiasm for epigenetics within evolutionary biology. An analysis of scientific abstracts from the past twenty-five years further reveals systematic differences among disciplines in their uses of the term *epigenetic*, especially with respect to heritability commitments and functional interpretations. Taken together, these results paint a nuanced picture of the rise of epigenetics and the possible neglect of transposon dynamics, especially among biomedical scientists.

## Introduction

It is widely maintained that biology is undergoing an epigenetic revolution. According to this narrative, the gene is being dethroned from its privileged explanatory and investigation-guiding roles. In its place, scientists are focusing on various epigenetic factors—equally significant to genes in their casual and information-bearing functions, or so it is argued—that have long been neglected in the study of development and evolution.

The study of human disease is one of the fields that epigenetics is expected to transform. Biomedical interest in epigenetics traces back to the discovery that widespread loss of DNA methylation is associated with cancer [1]. At the time, it was a significant discovery that cancer could be triggered not only by mutation in gene sequence, but also by the removal of methylation marks. During the 2000s, biomedical work on epigenetics explored the tendency for cells to acquire an elevated vulnerability to stress [2]. This phenomenon was associated with alterations to DNA methylation triggered by environmental factors, such as a reduction in quality of diet [3], that are potentially transmitted to offspring in utero [4]. More recently, we are seeing the rise of large-scale research consortia such as the Encyclopedia of DNA Elements (ENCODE), which seeks to identify all functional elements in the human genome by focusing in particular on “regions of transcription, transcription factor association, chromatin structure and histone modification” [5, p. 57]. ENCODE’s most controversial and widely publicized result states that over 80% of the human genome is associated with some biochemical function [5]. From a gene-centric perspective, this claim would be surprising, since protein-coding regions constitute a mere 4% of the human genome [6]. Detractors object that ENCODE’s finding relies on an overly permissive definition of function, that their study used unjustifiably weak criteria for identifying genetic candidates as functional, and that their framework cannot explain differences among species’ genome sizes [7–10]. In defense of ENCODE, some authors interpret their controversial statement as an estimate of the proportion of genomic regions that are of potential biomedical interest [11]. Generally speaking, it is clear that epigenetics has motivated considerable research within the biomedical sciences, challenging conventional notions of biological function and expanding the range of entities thought to be functionally relevant to human disease.

It is tempting to follow authors like Eva Jablonka and Marion Lamb, who claim that epigenetics involves a paradigm shift in biology [12], or Russell Bonduriansky and Troy Day, who suggest that epigenetics constitutes a “new understanding of inheritance and evolution” [13]. This is a seductive picture, especially to philosophers. Conceptual change in science is an established field of philosophical research. The study of gene concepts has been one of the most fecund topics within the philosophy of biology. This work reveals that scientific conceptions of the gene and genetic disease are in an ongoing historical dialogue with technological advances in biology [14–16]. To many philosophers, it would be unsurprising if further technological developments led to additional modifications to scientific conceptions of heredity. Gene concepts have proven to be fluid, the thinking goes. Why should gene centrism itself not be up for grabs?

Some authors challenge the suggestion that there is an epigenetic revolution afoot. It is possible to distinguish three general objections. The first takes issue with the claim that epigenetic insights qualify as revolutionary. Peter Godfrey-Smith notes that over the course of its historical development, molecular biology has become gradually less doctrinaire [17]. Theoretical principles that were central to this discipline in its early stages, such as G.W. Beadle’s one gene–one enzyme hypothesis, have become less important as molecular details have been filled in. Epigenetic phenomena might have posed a serious challenge to the principles on which molecular biology was founded. However, in Godfrey-Smith’s view, these phenomena are less threatening now that principles have been supplanted with mechanistic details.

A second objection focuses on the various meanings of ‘epigenetic’ [18–20]. Some instances of epigenetic regulation merely involve the (gene-mediated) influence of an environmental factor on some phenotype. Gene centrists have always allowed that environmental factors influence gene expression. Such examples of epigenetic phenomena are therefore not unorthodox. At the same time, the term *epigenetic* sometimes refers to the open-ended transmission of a phenotypic change that involves no change in gene sequence. This phenomenon is thought to be rare in eukaryotes [21], but would indeed call for a radical shift in biological thinking if it were common. Conflating familiar epigenetic effects with rarer or more controversial phenomena potentially gives a distorted impression of what the study of epigenetics is about.

A third objection concerns the functional interpretation of certain epigenetic phenomena. Epigenetic revolutionaries point to examples of phenotypic mutation that are induced by some environmental change, appear to be adaptive for the organism, and involve no change in DNA sequence, but are transmitted in sexual lineages across generations. Such examples are interpreted as evidence for a switch-like mechanism that rapidly adapts the phenotype to environmental change. This mechanism is allegedly less visible from a research program focused on genes. Also, if adaptive epigenetic inheritance is common, this challenges the neo-Darwinian idea that phenotypic adaptation typically involves random genetic variation and selection.

Our first aim in this paper is to explore an alternative explanation of epigenetic inheritance that views it not as an adaptive epigenetic switch, but rather as the byproduct of transposon dynamics. This explanation has long been available but is rarely considered, raising the question of whether transposon dynamics generally tend to be neglected in discussions about epigenetics. Our second aim is to address this question using a quantitative analysis of papers sampled from the Web of Science. In this way, we examine the popularity of *epigenetics* versus *transposons* across different disciplines over the past five decades. Finally, using a qualitative analysis comparing different conceptions of epigenetics across disciplines over the past twenty-five years, we compare the varied disciplinary views of epigenetics researchers on the topics of heritability and function.

### Epigenetic switches and the significance of transposons

One of the most widely discussed examples of epigenetic inheritance involves the transmission of coat coloration in lab mice. The *agouti* gene is expressed in mouse hair follicles and normally produces a dark brown coat. However, in some mice there is a change in the expression of this gene, producing a coat that appears sometimes yellow or on other occasions variegated. All strains of mice share an identical *agouti* gene with no variation in nucleotide sequence. Differences in coat color are instead produced by variation in methylation patterns upstream of the pigment gene. An interesting feature of this example is that color pattern is maternally inherited for up to three generations, indicating that parents transmit methylation patterns to their offspring.

The *agouti* gene has become a model system for epigenetics. For instance, a study by Dana Dolinoy et al. exposed female mice to bisphenol A (BPA) and noticed a shift toward yellow in the coat color distribution of their offspring [22]. Again, variation in coat color was caused not by a DNA mutation but rather by a change in methylation. Moreover, the effect was counteracted when female mice were fed a diet supplemented with methyl donors.

Such examples have been interpreted as evidence for an epigenetic inheritance mechanism, or switch, that rapidly adapts organisms to their environment. In discussing *agouti* gene expression in mice, Jablonka and Lamb propose:

Because it provides an additional source of variation, evolution can occur through the epigenetic dimension of heredity even if nothing is happening in the genetic dimension. But it means more than this. Epigenetic variations are generated at a higher rate than genetic ones, especially in changed environmental conditions, and several epigenetic variations may occur at the same time. Furthermore, they may not be blind to function, because changes in epigenetic marks probably occur preferentially on genes that are induced to be active by new conditions. [12, p. 144]

Likewise, Bonduriasnki and Day claim that the *agouti* mouse example “shows how such epigenetic traits could contribute to adaptive evolution” [13, p. 58]. There are three basic components to this interpretation. First, there is the proposal that phenotypic changes are induced by the environment. Second, there is the claim that those changes involve a modification to methylation or some other epigenetic mark, but no change in gene sequence. Finally, there is often the suggestion that epigenetic changes are biased toward adaptive phenotypic responses. The conjunction of these three propositions is what we refer to as the epigenetic switch hypothesis.

Others have raised doubts about the existence of epigenetic switches because the relevant effects persist for no more than three generations. To be of evolutionary interest, it is argued, an epi-mutation would have to persist for much longer. A recent review by Alfredo Sánchez-Tójar et al. found little evidence for such transgenerational epigenetic effects. However, this remains a topic for further research [21].

Perhaps a more philosophically interesting objection concerns the fact that the *agouti* mutation involves the suppression of a transposable element, located upstream of the *agouti* gene. Jablonka and Lamb mention in passing that “there was a small extra bit of DNA (originating from a transposon) in the regulatory region of a coat color gene” [12, p. 142]; but they overlook the theoretical significance of this point. As we explain in the next few paragraphs, the fact that epigenetic mutations are often transacted by transposable elements suggests an alternative to the epigenetic switch hypothesis.

Transposable elements (TEs) are mobile strands of DNA capable of jumping into new chromosomal locations. The act of transposition (jumping) often involves the creation of additional TE copies. Hence, individual TEs can replicate multiple times per generation in a process akin to meiotic drive. It is well known that TE insertion can interfere with protein synthesis or cause various sorts of harmful mutation. Organisms have thus evolved a variety of mechanisms for deactivating, suppressing, or removing TEs from the genome. These mechanisms, in turn, impose a selection pressure on TEs to evolve ways to overcome the host organism’s defenses. Over millions of years, these coevolutionary dynamics have given rise to eukaryotic genomes replete with TEs—with 40–60% of the nuclear DNA in humans descending from TEs—most of which are temporarily silenced or permanently deactivated [23].

There are several reasons TEs may appear to have organism-beneficial functions when they are in fact deleterious. One way for a TE lineage to potentially avoid deactivation or deletion is by inserting copies very close to a protein-coding gene [24]. These sites are safe havens, so to speak, because the host cannot easily methylate TEs at these locations without altering the expression of its own genes. It is therefore no surprise that many TEs preferentially insert close to protein-coding genes [25].

It is easy to mistake these stealthy TEs for organism-beneficial insertions [10]. Genomics researchers identify the strands of DNA located adjacent to genes as regulatory regions because they contain transcription factor binding sites. The occurrence of TEs within regulatory regions has led some genomics researchers to implicate them in gene regulation, disregarding the possibility that the TEs might simply be hiding in a safe location. This interpretation is further supported by the fact that TEs contain their own binding sites which are normally used to harness the host’s replication machinery for their own benefit. Hence, TEs are especially effective mimics of genuine regulatory regions.

Another deceptive feature of TEs is that they are activated by stress. When an organism is exposed to chemical, thermal, or other forms of stress, there is sometimes a burst of TE activity [25]. Barbara McClintock has interpreted TE bursts as evidence for a switch-like mechanism that facilitates rapid phenotypic adaptation by elevating mutation rate [26]. Once again, however, the situation looks different from the perspective of TE–host coevolution. Organisms employ various strategies to protect genes from TE insertion. Some suppression strategies occur at the level of the DNA strand, where methyl groups are inserted on top of transposon binding sites to prevent them from being recognized by the host’s transcription factors and replicated. In fact, it is now thought that DNA methylation originated as a system for TE suppression, with gene regulation a secondary (exapted) function [27]. Important for our argument is that suppression mechanisms are themselves compromised by stress. Just as a parasite can get the upper hand on a patient with a compromised immune system, so can TEs flourish in a genome with weakened suppression. Thus, what appears to be switch-like behavior in response to environmental change might in fact be a breakdown in TE suppression machinery.

These considerations cast new light on the *agouti* mouse example. Recall that variability in coat color is caused by variable methylation patterns surrounding TE insertion upstream of the pigment gene. It is quite plausible that different color morphs represent different levels of TE suppression, with more heavily methylated strains being a step ahead in the coevolutionary arms race. Were this TE to degrade or be removed, the site would presumably cease to become hyper-methylated and the yellow phenotype would disappear. Moreover, if this example is typical, and epigenetic effects typically involve an effort to suppress TEs, then it is unlikely that epigenetic mutations will have adaptive effects. It is essentially up to the transposon to determine where it wants to insert. Selection acting among TE lineages (within the organism) will favor transposons that avoid detection and deletion. This might involve stealthy insertions close to genes in some cases or in other cases the avoidance of genic regions altogether, but there is no reason to expect an insertion preference for regions that will benefit the host.

David Haig argues that it is often in the evolutionary interest of both the organism and the transposon for TE insertions to be silenced in somatic tissues (as opposed to the germ line) [28]. This allows the host organism to survive and reproduce, passing along its complement of TEs to the next generation. Evolutionary interests conflict more directly in the germ line. If a TE insertion kills the host, then the TE will be removed from the population. This imposes a downward selection pressure moderating the rate of TE replication. However, it has long been recognized that in sexual species it is difficult for selection to entirely purge the genome of determinantal TEs [29]. Eukaryotic organisms are stuck with these genetic parasites and, again, there is no reason to expect that TEs will preferentially insert into regions that are likely to benefit the host. Nor does the methylation of those insertions occur with some directed beneficial effect on the organism, other than to mitigate the negative effects of a TE insertion on normal host function. These considerations cast doubt on the idea that epigenetic responses to environmental change will tend to be adaptive, at least, not insofar as they are associated with the suppression of TEs.

If epigenetic differences are typically driven by responses to TE insertion, this also has implications for the persistence of epi-mutations. Organisms are engaged in a constant effort to detect and suppress TEs. Eventually, active TE insertions will degrade and no longer attract methylation. As a result, any TE-mediated switch will have a limited life span because processes within the organism are actively degrading it.

What about the suggestion that epigenetic switches respond to specific environmental cues? From a coevolutionary perspective, not just any environmental factor can be “hooked up” to the epigenetic machinery. If the loss of methylation is typically caused by a breakdown in TE suppression, then only harmful environmental factors will induce this type of epigenetic change. Relatedly, after the stressful conditions have subsided, the TE suppression machinery ought to resume its job of methylating TE insertions. Hence, unless the organism is exposed to a continual regime of stress, persisting over many generations, one would expect TE-based epigenetic mutations to be short lived.

The topic of TE–host dynamics is a fascinating area of research that would take us beyond the objectives of this paper to describe in detail. We hope to have said enough to at least raise questions about the ways that examples of epigenetic inheritance are interpreted by some proponents of the epigenetic switch hypothesis. At the very least, one might expect that considerations about TE dynamics would be raised as an alternative explanation for examples such as *agouti* gene expression in mice. Instead of being viewed as an epigenetic switch, the environmental induction and epigenetic transmission of the colored phenotype might simply be the byproduct of TE suppression. Why has this alternative been largely ignored by authors working on epigenetic inheritance?

It has been suggested that the fields of molecular biology and genomics are simply out of touch with recent trends in evolutionary biology [30]. This could be due to insufficient evolutionary training in those fields. Another potentially relevant factor is the high prevalence of adaptationist thinking within molecular biology and genomics. A number of authors have noted that adaptationist hypotheses are unjustifiably popular in these disciplines [7–9, 31, 32]. Another, non-exclusive possibility concerns the influence of large research consortia like ENCODE and the economic incentives driving these projects. Garnering large sums of public funding sometimes involves interpreting results in ways that sound exciting, revolutionary, or relevant to human disease. Describing examples like the *agouti* mouse coat coloration as an epigenetic switch sounds more exciting than the alternative possibility, that this phenomenon is the fleeting, stress-induced byproduct of a genetic parasite.

We have suggested that information about TE–organism coevolution recommends an explanation of certain epigenetic phenomena that rivals the epigenetic switch hypothesis. This raises the question of whether, given the ballooning popularity of epigenetics research, those coevolutionary dynamics are generally being overlooked or downplayed. This question can be explored by comparing the relative popularity of epigenetic versus transposon research over time and across disciplines. We expect that researchers working in the field of evolution, who are familiar with genome-level coevolutionary dynamics, are less enthusiastic about epigenetics compared to researchers working in proximal biological sciences, where evolutionary thinking is less common. Likewise, if the attraction to epigenetics is influenced in part by large research consortia like ENCODE, then one might expect epigenetics to be more popular in biomedical biology and genomics compared to other disciplines.

A related set of questions concerns the ways that different disciplines conceptualize epigenetics. It is possible that researchers in biomedical fields rarely embrace the epigenetic switch hypothesis and use *epigenetic* to refer to different phenomena than researchers working in other disciplines, for instance. The remainder of this paper describes two bibliometric studies attempting to shed light on these questions.

## Methods

### Topics and disciplines

Our methods were inspired by Haig’s survey of scientific articles published between 1950 and 2010, which shows a dramatic increase in the proportion of scientific papers with *epigenetics* in the title [19]. Using digital tools and databases associated with the Web of Science, we undertook two bibliometric analyses of scientific articles. The Web of Science platform allows users to search for papers containing terms in specific fields (e.g., title, keywords, or associated metadata). We first selected all papers in the Web of Science published in English between 1970 and 2019 that contain *DNA* in their topic field—which subsumes the title, abstract, author, and keywords fields—and organized them into five-year intervals. To give some sense of the results, between 1970 and 1974, there were roughly 10,000 papers published on DNA. By 2015–2019, there were over 315,000 papers on this topic. We then selected the subset of DNA papers that also contain *epigenetic* as a root word in their topic field and repeated this procedure for *transposon/TE/transposable element* as a root word. Considering that the Web of Science is a comprehensive citation catalogue, our analyses likely include the majority of scientific papers published on the subject of DNA. As a result, scientific interest in epigenetics and transposons can be compared as proportions of the total scientific interest in DNA over time. Although the absolute number of papers on any topic will tend to increase given the growing number of scientific articles published each year, the proportion of papers on a topic will either rise or fall depending on its popularity. Hence, our measure provides an estimate of the proportional interest in epigenetics and transposons.

Journals in the Web of Science are assigned codes according to subject, known as the Web of Science Subject Categories, and all papers appearing within a given journal are allocated to its corresponding category or categories. When searching *epigenetics* within *DNA*, there are hundreds of categories ranging from genetics and heredity to logic to theatre. However, most papers fall within a small number of categories. We focused our analysis on what we identify as four disciplines: biomedicine, proximal biology, evolution, and general biology. Biomedicine is a conjunction of five Web of Science categories: medicine general internal, medicine research experimental, oncology, pharmacology, and immunology. These were chosen partly because they are highly represented and partly because they fall under the general theme of biomedical research. We lumped them into a single variable primarily to simplify the analysis. However, we performed a consistency check, comparing each category within the biomedical discipline to check for anomalies in their relative proportions.

Likewise, proximal biology is a conjunction of four Web of Science categories: cell biology, developmental biology, genetics heredity, and biochemistry molecular biology. Again, these categories are highly represented under the topic of DNA, and they exhibit a number of thematic similarities. We applied the same rationale and consistency check to these categories.

General biology and evolution are stand-alone categories provided by the Web of Science. We included general biology in our analysis with the expectation that it would provide a baseline for comparing other disciplines. Evolution was included because of its relevance to our focal questions.

### Quantitative analyses

We conducted two quantitative analyses to determine relative scientific interest in epigenetics and transposons across disciplines. The first analysis tracks the proportion of papers on epigenetics in the broader pool of publication on DNA within each of the four disciplines across all five-year intervals. The second analysis does the same for papers on transposons. These analyses together provide a gauge of relative scientific interest in these two topics among the four disciplines over the past fifty years.

### Epigenetic commitments

It is widely recognized that the term *epigenetic* is ambiguous, and it is rarely possible to glean a definition of this term from a research paper. However, it is usually possible to discern certain logical commitments based on what authors say about epigenetic phenomena. For the purposes of our analysis, we propose two dimensions along which such commitments can be seen to vary. The first dimension involves authors’ heritability commitment. In classifying some modification to DNA as epigenetic, one might simply be referring to a basic mark (e.g., a methylation pattern or histone modification) that is conspicuously associated with DNA. Minimally, there need be no commitment as to whether that mark is inherited by daughter cells or for how long. A stronger commitment maintains that epigenetic marks are transmitted mitotically when cells divide, but remains agnostic about transmission by meiosis. A third level of commitment proposes limited meiotic cell division, such as when an epigenetic mark is transmitted to offspring but no further. Finally, the strongest commitment proposes open-ended meiotic transmission. This is the level of commitment that is often associated with the epigenetic switch hypothesis. These definitions are summarized and operationally defined in Table 1.

**Table 1:** Four conceptions of epigenetic phenomena with varying strengths of heritability commitments

Inheritance	Commitment	Operational definition
Basic mark	The presence or absence of some mark is associated with DNA (e.g., methylation, histone modification), but its heritability is unspecified	Applied to abstracts describing differences in epigenetic factors over time or comparing epigenetic similarities/differences among cells, but making no explicit mention of whether those factors are heritable
Mitotic inheritance	Some mark associated with DNA is transmitted by mitotic cell division	Applied to abstracts explicitly mentioning mitotic transmission and/or describing the persistence throughout division in a somatic cell lineage
Limited meiotic inheritance	Some mark associated with DNA persists through meiotic cell division and/or is transmitted over a limited number of sexual generations	Applied to abstracts explicitly mentioning epigenetic transmission from parent to offspring up to the F2 generation
Open-ended inheritance	Some mark associated with DNA persists indefinitely through meiotic cell division and/or is transmitted over a large number of sexual generations	Applied to abstracts explicitly proposing a multi-generational epigenetic influence (e.g., “transmitted over many generations”) or equating the heritability of epigenetic marks with genes. This level of commitment is often associated with the epigenetic switch hypothesis

The second dimension concerns authors’ functional interpretation of epigenetic marks. None of the heritability commitments just outlined implies that an epigenetic mark is functional. We think it is crucial not to conflate heritability commitments with functional interpretations because both have different epistemic criteria. Advances in sequencing technology have greatly simplified the ability to detect epigenetic marks and their varying degrees of heritability. As the ENCODE controversy reminds us, assessing function is much more difficult and often contentious. A related concern is that if function is conflated with inheritance, researchers might gravitate toward a particular functional interpretation without demanding adequate evidence. Carrie Deans and Keith Maggert note that this is in fact a common mistake: “It’s not that histone modification and DNA methylation are not correlated with gene expression differences—they are—but the possibility that they may be responsive rather than causal has not been disproved” [33]. The list of functional roles analyzed in our study are outlined in Table 2.

**Table 2:** Four functional roles commonly associated with epigenetic marks

Functional role	Explanation	Operational definition
Disease relation	Some epigenetic mark associated with a disease (e.g., tumor growth) is thought to influence its promotion or suppression	Applied to abstracts implicating an epigenetic mark in some disease, but not to those explicitly proposing that the mark is involved in normal gene expression or phenotypic development
Transposon suppression	Some epigenetic mark is thought to normally function in the suppression of TE activity	Applied to abstracts explicitly assigning this functional role to epigenetic marks, but not to those proposing that TE activity is part of an epigenetic mechanism for adaptive phenotypic plasticity
Regulation	Some epigenetic mark is thought to normally function in the regulation of a gene and/or trait	Applied to abstracts proposing that an epigenetic mark regulates gene expression or trait development
Phenotypic adaptation	Some epigenetic mark is thought to regulate genes in ways that adapt the organism to its environment, either by adaptively modifying the phenotype to environmental changes or by stabilizing a beneficial phenotype	Applied to abstracts explicitly proposing that epigenetic marks preserve adaptive phenotypes or suggesting that they function in adaptive phenotypic plasticity. This interpretation is often associated with the epigenetic switch hypothesis

### Qualitative analyses

To compare heritability commitments and functional interpretations across disciplines over time, we focused on the top twenty-five most cited papers about epigenetics under the topic of DNA for each five-year interval from 1995 to 2019. The reason for not going back further is that one of the Web of Science categories (evolution) has fewer than twenty-five papers per five-year period prior to 1995 and would have biased our comparisons. To categorize the heritability commitments and functional interpretations of each paper, we carefully examined each title and abstract and classified the paper according to the operational definitions outlined in Tables 1 and 2. Only one heritability commitment and one functional interpretation was assigned to each article.

## Results

**Fig. 1:**
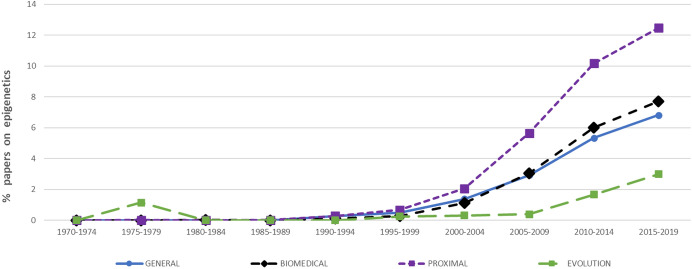
Percentages of papers in the Web of Science on the general topic of DNA mentioning epigenetics in the title, abstract, or keywords, viewed in five-year intervals across four biological disciplines

Our quantitative results are consistent with the trend reported by Haig [19]: there is a sharp rise in the proportion of epigenetics papers beginning in the mid-1990s (Fig. 1). In the most recent interval (2015–2019), there were in total 316,191 papers on the topic of DNA. Within just this part of our sample, looking only at our four focal disciplines, a whopping 19% mentioned epigenetics in the title, keywords, or abstract. However, there was considerable variation among disciplines over time in their enthusiasm for the topic. Proximal biology is an early adopter, with biomedicine and general biology showing a more delayed response. By contrast, the delayed and relatively small amount of enthusiasm coming from evolution is striking. This discipline begins warming to epigenetics only after 2005, and its contribution to the pool of papers on epigenetics remains low.

**Fig. 2:**
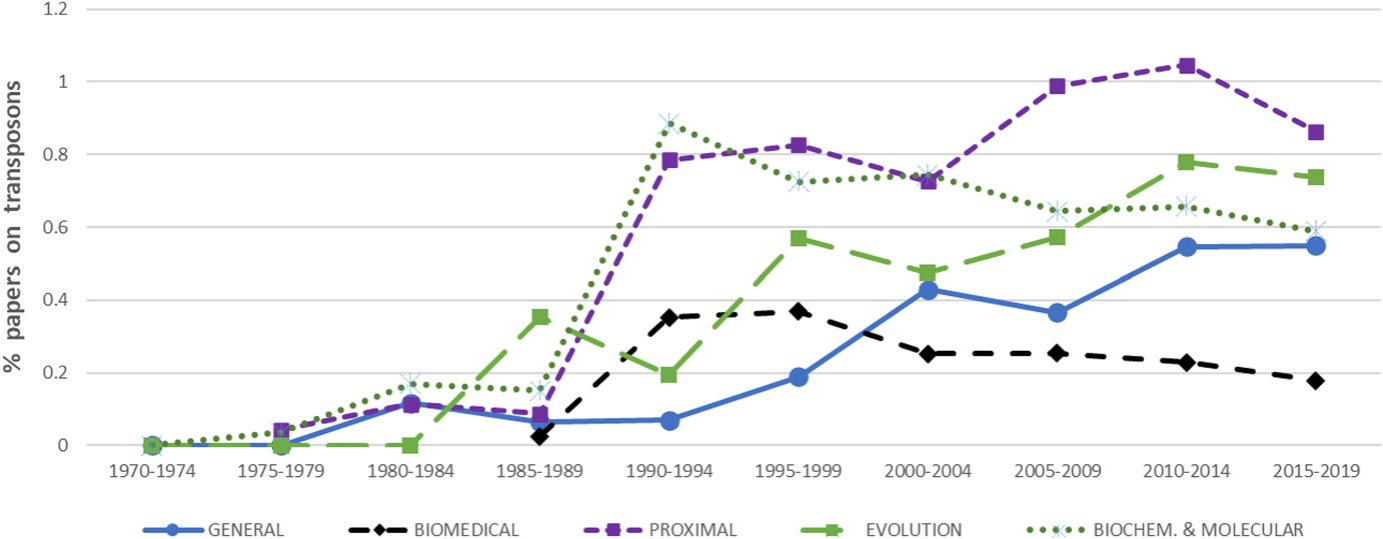
Percentages of papers in the Web of Science on the general topic of DNA mentioning transposons in the title, abstract, or keywords, viewed in five-year intervals across four biological disciplines (plus biochemical and molecular biology, a subdiscipline of proximal biology)

Compared to epigenetics, overall enthusiasm for the topic of transposons is relatively low, never exceeding 2% of the total papers on DNA. Across all disciplines there is a spike in transposon research beginning in the early to mid-1980s (Fig. 2). Evolution and general biology show steady increases in the proportions of papers on transposons. By contrast, biomedicine initially shows interest in transposons in the late 1980s and early 1990s, but this interest tapers in the late 1990s and starts declining in the early 2000s.

The pattern exhibited by proximal biology is more complicated. Interest in transposons picks up in the late 1980s, flattens during the 1990s, and picks up again in the early 2000s. Only in the last five years has interest in transposons started to decline in proximal biology. Our consistency check revealed a divergence among the categories comprised by this discipline. Within cell biology, developmental biology, and genetics heredity, the proportional interest in transposons begins to decline only in the last five years. However, in the field of biochemistry and molecular biology, the decline begins much earlier and follows a pattern similar to biomedicine.

**Fig. 3:**
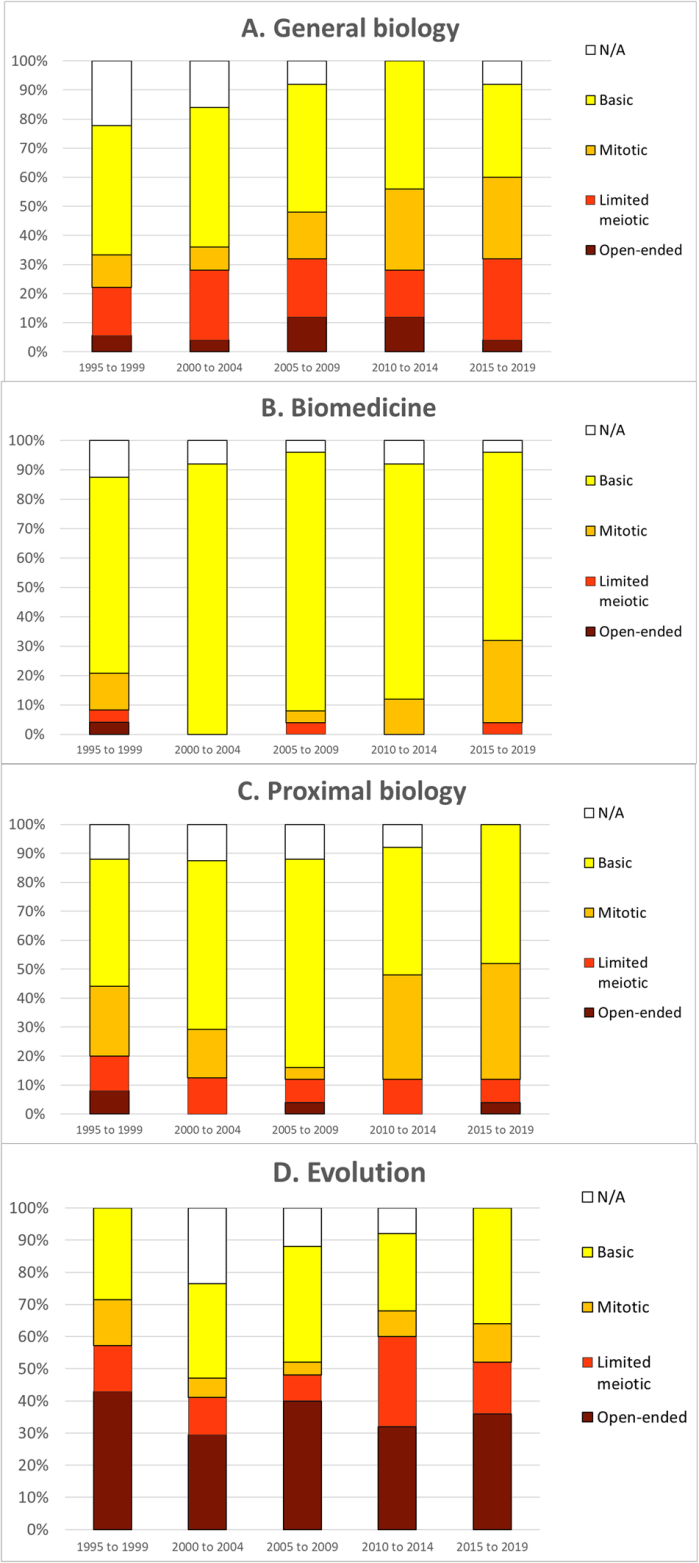
Breakdown of heritability commitments reflected in abstracts of twenty-five most cited articles on DNA/epigenetics in the Web of Science per five-year interval in **(A)** general biology, **(B)** biomedicine, **(C)** proximal biology, and **(D)** evolution

Turning to our qualitative analysis of heritability commitments, in analyzing these data we were interested in whether a particular commitment is dominant in a given discipline and whether the disciplinary prevalence of commitments changes over the twenty-five-year period. The results reveal that general biology exhibits a broad mixture of heritability commitments, as might be expected if this discipline is regarded as a baseline (Fig. 3a). A large and stable percentage of papers across the entire period (32–42%) make basic reference to epigenetic marks without specifying heritability. There appears to be a slightly growing trend in commitments to mitotic inheritance, from 8 to 10% of papers in the 1990s to 25–28% in the most recent decade. Commitments to limited meiotic inheritance have remained stable at 20–30%, with a slight dip to 7% between 2010 and 2014. The least common commitment is to open-ended inheritance, accounting for 5–10% of papers throughout.

Biomedicine exhibits a simpler pattern, with a dominant majority of papers across the entire period (more than 60%) referring to epigenetic marks of indeterminate heritability and a slight increase in commitments to mitotic inheritance over the last decade (Fig. 3b). The majority of papers in proximal biology also refer to epigenetic marks of indeterminate heritability, ranging from 42 to 72% (Fig. 3c). However, in the two most recent intervals, commitments to mitotic inheritance have been roughly equal to commitments to bare marks (32–39%). In both biomedicine and proximal biology, commitments to limited meiotic inheritance are quite infrequent (consistently less than 10%), with almost no papers committing to open-ended inheritance. This trend is in sharp contrast to that seen in evolution, where open-ended inheritance is the most popular commitment, ranging from 42% of papers in the 1990s to around 33% of papers in the most recent decade (Fig. 3d). The next most common commitment in evolution is to basic epigenetic marks with unspecified heritability (consistently 25–35%). The discipline has shown a slight increase in commitments to limited meiotic inheritance in recent years, but very few commitments to mitotic inheritance.

**Fig. 4:**
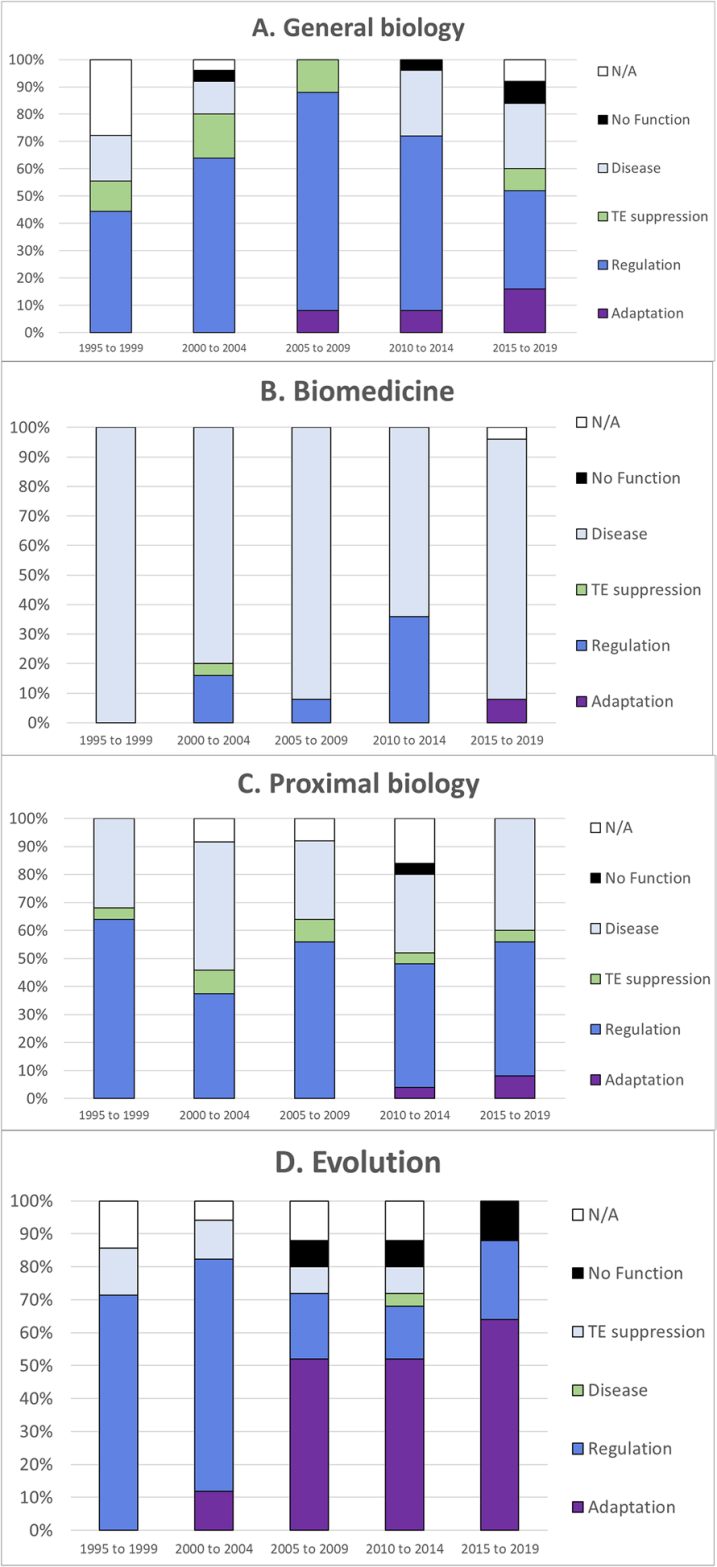
Breakdown of functional interpretations of epigenetic marks reflected in abstracts of twenty-five most cited articles on DNA/epigenetics in the Web of Science per five-year interval in **(A)** general biology, **(B)** biomedicine, **(C)** proximal biology, and **(D)** evolution

Now turning to our second qualitative analysis, in analyzing these data we were interested in whether a particular functional interpretation is dominant in a given discipline and whether the disciplinary prevalence of functional interpretation changes over the twenty-five-year period. Within general biology regulation is the dominant functional interpretation of epigenetic marks (Fig. 4a). However, this interpretation seems to peak in the early 2000s, when it accounts for 80% of papers, falling to 35% in the most recent interval. In biomedicine, it is perhaps no surprise that the most common functional interpretation is relevant to disease, with disease-related functions represented in over 80% of papers for all but one interval, 2010–2014, when functional interest in regulation briefly spikes to 37% (Fig. 4b). By contrast, in proximal biology, regulation (38–61%) and disease (24–36%) are the two most popular functional interpretations (Fig. 4c). Interestingly, there is a low but persistent interest in TE suppression (2–7%) among proximal biology papers across the entire period. Evolution again diverges from other disciplines. Here there is a shift from a majority interest in regulation, roughly 70% in 1995–2004, to a majority interest in adaptation, growing from 52% in 2005–2009 to 86% in 2015–2019 (Fig. 4d). Evolution is the only discipline that shows such a sharp and dramatic swing in the prevalent functional interpretation of epigenetic markers.

## Discussion

Our analyses in this study were motivated by the question of whether transposon dynamics are neglected by researchers interested in epigenetics generally, as they seem to be by some proponents of the epigenetic switch hypothesis. TE coevolutionary dynamics have been largely understood since the mid-1980s. So when it comes to examples like the *agouti* mice, where phenotypic effects are caused by methylation of a known TE insertion, one might expect researchers to entertain TE dynamics as a viable alternative to the presence of an epigenetic switch. Yet such consideration is somewhat rare.

It has been suggested that the disciplines of molecular biology and genomics are out of touch with advances in evolutionary theory [8, 9, 30]. If this is correct, then one would expect to see less enthusiasm for epigenetics in evolution than in proximal biology or biomedicine. This prediction is borne out in Fig. 1, where evolution shows a delayed and relatively muted interest in epigenetics compared to other disciplines. Figure 2 exhibits declining interest in transposons in biomedicine and in molecular biology, though not in the other categories comprised by proximal biology (cell biology, developmental biology, and genetics). It is difficult to understand why, as TEs are increasingly recognized as major constituents of eukaryotic genomes, and given their known mutagenic effects, the biomedical sciences are gradually becoming less interested in transposable elements. At the very least, one would expect an increased interest both in epigenetics and in transposons in biomedicine, as with the other disciplines in our sample.

What explains evolution’s delayed and relatively muted interest in epigenetics compared to other disciplines? Believers in the epigenetic revolution might take this reticence to suggest that evolution is a conservative discipline, clinging to the dogma of gene centrism. Alternatively, the discipline’s greater familiarity with transposon dynamics and genome evolution might mean that its practitioners are simply less enamored by functional interpretations that ignore these factors. Likewise, the limited influence of large-scale funding organizations on evolution compared to biomedicine and proximal biology might also explain the differential enthusiasm for epigenetics across these disciplines. For whatever reason, evolutionary thinkers have been slower to jump on the epigenetic bandwagon. Perhaps questionnaire methods could help to answer the finer-grained question of why exactly this is the case.

It should be kept in mind that evolution papers on epigenetics have embraced a different, generally stronger set of heritability commitments and functional interpretations than similar papers in proximal biology, biomedicine, and to some extent general biology—frequently positing open-ended or limited meiotic transmission of marks and increasingly interpreting the function of epigenetic phenomena in terms of phenotypic adaptation. Putting these findings together, one could say that although the topic of epigenetics is relatively unpopular in evolutionary circles, those thinkers who do embrace epigenetics are more extreme in both their heritability commitments and functional interpretations. Also noteworthy is the sea change in functional interpretations that occurs in the mid-2000s, away from basic gene regulation and toward adaptive responses to environmental changes. This coincides with the publication of Jablonka and Lamb’s influential book [12] and could reflect its impact on evolutionary thinking.

It is noteworthy that biomedicine and proximal biology largely overlap in their heritability commitments and adopt quite similar functional interpretations. Despite the general concern that epigenetics research is fraught with ambiguity [20, 33], our analysis suggests that at least in these disciplines, authors mean roughly the same thing by “epigenetic.” The same can be said neither for general biology, where there is much more diversity in heritability commitments and functional interpretations, nor of course for evolution.

In sum, our results support the suspicion that interest in transposons is not just overshadowed by enthusiasm for epigenetics; rather, in some fields where enthusiasm for epigenetics is most prevalent (biomedicine, biochemistry, and molecular biology), interest in TE dynamics is actually on the decline. We suspect that this trend could lead researchers in these disciplines to uncritically embrace certain functional interpretations, such as the epigenetic switch hypothesis, without due consideration of alternative explanations. We hope that our findings will inspire further interest in transposon dynamics, especially among researchers drawn to the idea of an epigenetic revolution.
